# Supplementary material for: Integrated wafer-scale ultra-flat graphene by gradient surface energy modulation
Source: Nat Commun. 2022 Sep 15;13:5410. doi: 10.1038/s41467-022-33135-w (PMC9477858; doi:10.1038/s41467-022-33135-w)
Supplement: Supplementary file 1 — Supplementary Information [file 41467_2022_33135_MOESM1_ESM.pdf]

Supplementary Information

Integrated wafer-scale ultra-flat graphene by gradient surface energy modulation

Xin Gao[†], Liming Zheng[†], Fang Luo[†], Jun Qian[†], Jingyue Wang, Mingzhi Yan, Wendong Wang, Qinci Wu, Junchuan Tang, Yisen Cao, Congwei Tan, Jilin Tang, Mengjian Zhu[*], Yani Wang, Yanglizhi Li, Luzhao Sun, Guanghui Gao, Jianbo Yin, Li Lin, Zhongfan Liu, Shiqiao Qin[*], Hailin Peng[*]

Correspondence to: hlpeng@pku.edu.cn, sqqin8@nudt.edu.cn, zhumengjian11@nudt.edu.cn

This PDF file includes:

Section I: Step-by-step protocol of GSE transfer method (Supplementary Figure 1-3, Supplementary Movie 1)

Section II: Surface energies of different surfaces (Supplementary Figure 4, Supplementary Table 1-2)

Section III: The Gradient surface energy (GSE) strategy enables the intact and clean transfer of ultra-flat wafer-scale graphene (Supplementary Figure 5-14)

Section IV: The universality of GSE strategy (Supplementary Figure 15-18, Supplementary Movie 2)

Section V: Raman spectra of GSE-transferred graphene (Supplementary Figure 19)

Section VI: Electrical performance of transferred graphene on SiO_2/Si (Supplementary Figure 20-22)

Section VII: Morphology and Raman peak position shift of graphene thermal emitter devices (Supplementary Figure 23)

Supplementary References (1-22)

Section I: Step-by-step protocol of GSE transfer method (Supplementary Figure 1-3, Supplementary Movie 1)

The images of crucial steps of GSE transfer method are shown in Supplementary Figure 1.

1. A layer of (-)-borneol dissolving in isopropyl alcohol (25 wt%) and a layer of PMMA were sequentially spin-coated on graphene/Cu(111)/sapphire at 1000 rpm for 1 min, and baked at 130 °C for 3 min to form a composite support film.
2. PMMA/Borneol/graphene film was detached from growth substrate by etching the Cu film in an aqueous solution of 1 mol L^{-1} $(NH)_4S_2O_8$, and etching was completed in 8~12 h for 4-inch graphene/Cu(111)/sapphire wafer. In addition, the detachment time can be shortened to ~5 min by electrochemical bubbling method, where PDMS/PMMA/borneol was directly used as a composite support layer.
3. After washing with deionized water to remove residual etchant, the PMMA/Borneol/graphene film was scooped onto the SiO_2/Si substrates, and the PDMS was laminated on the surface of PMMA/borneol when the graphene was fully dried using the commercial laminator, details can be seen in Supplementary Movie 1.
4. The composite film of PDMS/PMMA/Borneol/Graphene was peeled off from SiO_2/Si in water because the water will intercalate into the interface of graphene and SiO_2/Si due to the hydrophilic surface of SiO_2/Si when PDMS/PMMA/borneol/graphene/SiO_2/Si immersed in water (Supplementary Figure 2 and Supplementary Movie 1).
5. The composite film is fully dried in atmosphere, followed by laminating onto SiO_2/Si (sapphire), and the PDMS was exfoliated from the PMMA at 180 °C in 5 min (Supplementary Movie 1).
6. To further enhance the interaction of graphene and substrate, we baked the graphene at 180 °C for 3 h before removing PMMA and borneol with the vapor of hot acetone, leaving the monolayer graphene on target substrate. The hot vapor of acetone was used to remove PMMA/borneol by heating liquid acetone to the boiling temperature. The vapor of acetone can remove most PMMA and drop into the liquid acetone. Then, fresh acetone vapor will rise and further remove the PMMA and borneol residues on the graphene surface.

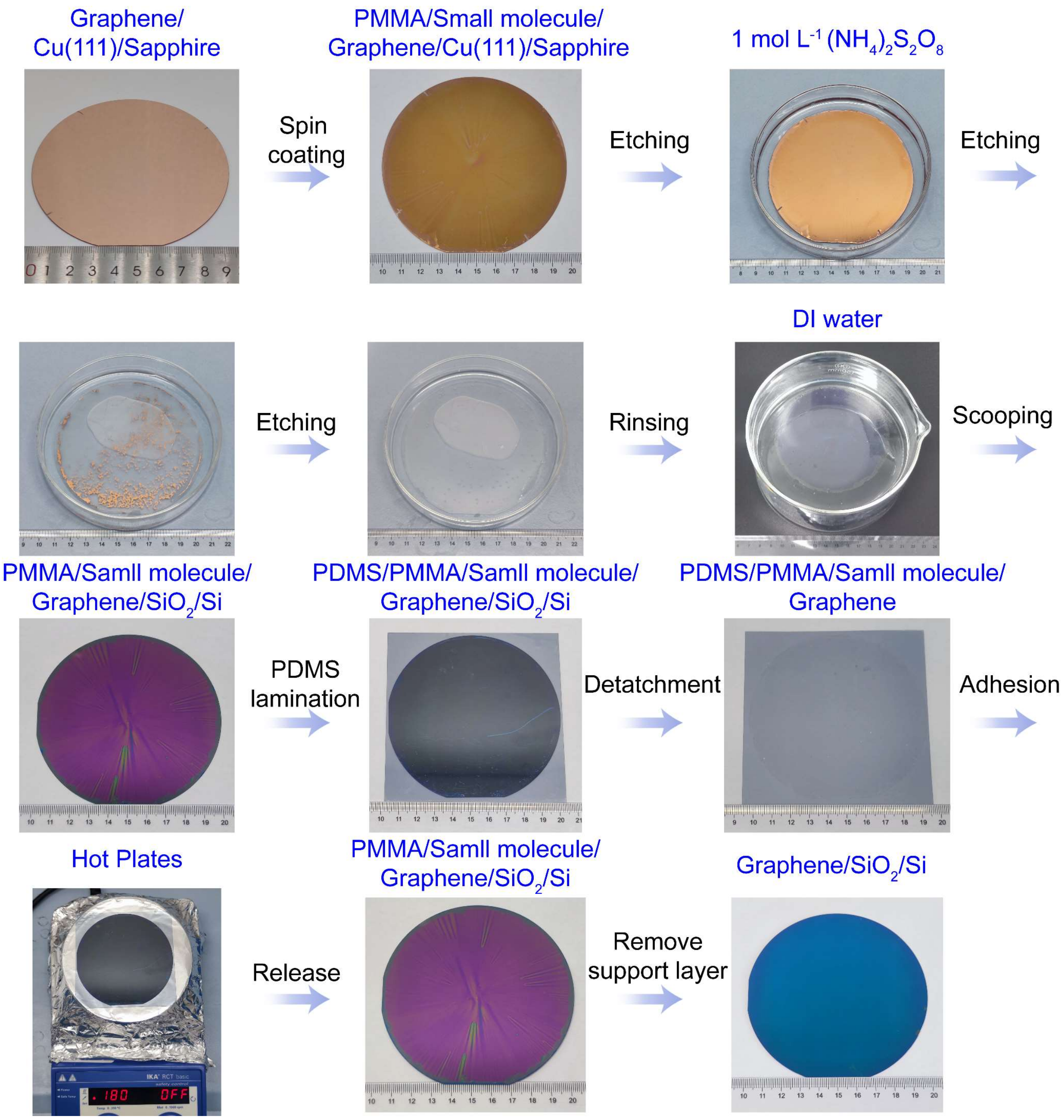

Supplementary Figure 1. The details of GSE transfer process.

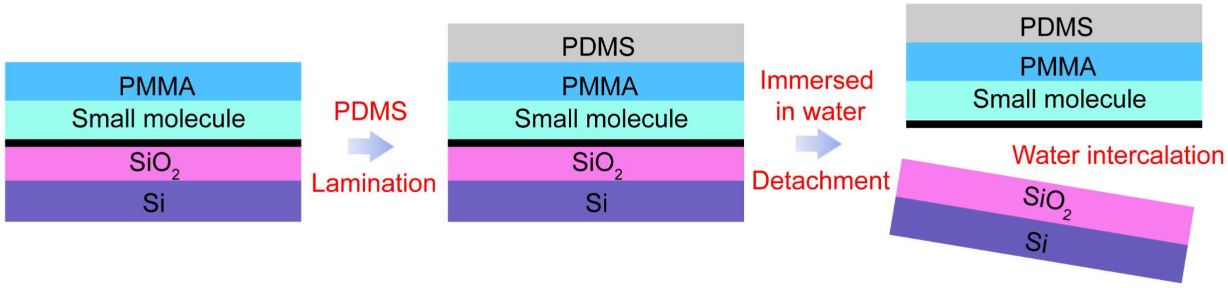

Supplementary Figure 2. Details of GSE transfer process. The lamination of PDMS and detachment of PDMS/PMMA/borneol/graphene composite film. The detachment was assisted by water intercalation.

The water will intercalate into the interface of graphene and SiO_2/Si due to the hydrophilic surface of SiO_2/Si when PDMS/PMMA/borneol/graphene/Si immersed in water. Then the composite film can be peeled off from substrate as shown in Supplementary Figure 2 and Supplementary Movie 1. The PDMS of obtained composite film can serve as a self-supporting layer, allowing the dry transfer of graphene to versatile wafers and preventing water-adsorption-induced p-doping.

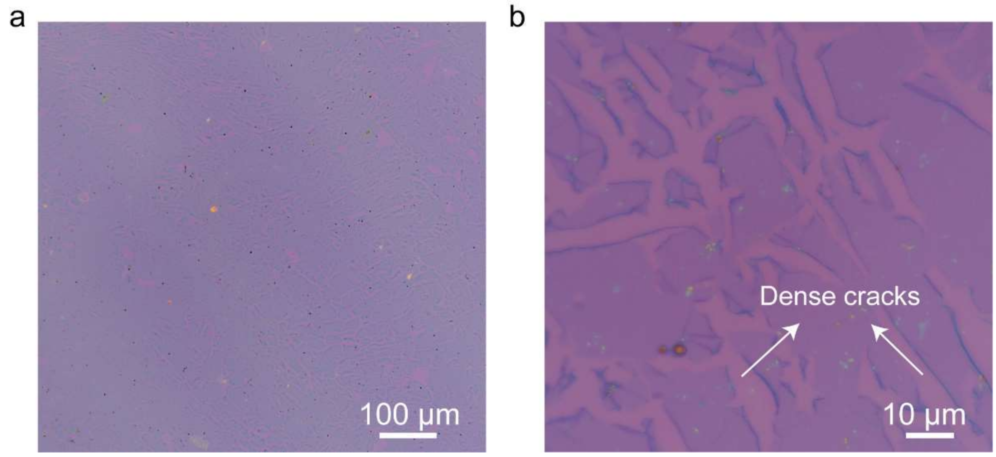

Supplementary Figure 3. Optical microscopy images of transferred graphene without PMMA support layer. (**a**-**b**) Optical microscopy images of PDMS-transferred graphene with 10× and 100× objective, respectively.

Note that the borneol layer is rather fragile, so the PMMA layer is designed to support the borneol/graphene and ensures the integrity of wafer-scale graphene during transfer. If the PMMA layer is not used in the transfer medium, the obtained graphene film will suffer from dense cracks (Supplementary Figure 3).

Section II: Surface energies of different surfaces (Supplementary Figure 4, Supplementary Table 1-2)

The surface energies (Supplementary Table 1) were calculated by measuring the contact angles of water and glycerol based on Owen-Wendt (1) and Young's equation (2)[1]. Equation (3) was obtained by combing equation (1) and (2).

$$\gamma_{sl} = \gamma_s + \gamma_{lv} - 2\left(\gamma_s^d \gamma_{lv}^d\right)^{\frac{1}{2}} - 2\left(\gamma_s^p \gamma_{lv}^p\right)^{\frac{1}{2}} \quad (1)$$

$$\gamma_s = \gamma_{lv} \cos\theta + \gamma_{sl} \quad (2)$$

$$\gamma_{lv}\left(\cos\theta + 1\right) = 2\left(\gamma_s^d \gamma_{lv}^d\right)^{\frac{1}{2}} + 2\left(\gamma_s^p \gamma_{lv}^p\right)^{\frac{1}{2}} \quad (3)$$

γ_{lv} and γ_s are the surface energies of used liquid and solid surface, respectively; θ is the contact angle of tested liquid on the substrate; d and p are the dispersion and polar components, respectively. Water and glycerol were used as tested liquids with surface energies listed in Supplementary Table 2. The images of water and glycerol on different surfaces are shown in Supplementary Figure 4.

Supplementary Table 1. Surface energies and contact angles of different surfaces

Substrate	Contact angle(°)		Surface energy (mN/m)
	Water	Glycerol	
SiO_2/Si	26.6 ± 1.9	14.8 ± 1.2	65.9 ± 2.0
Graphene/Borneol	87.3 ± 3.0	75.5 ± 3.1	26.5 ± 2.8
PMMA	101.1 ± 1.3	90.6 ± 1.7	17.1 ± 2.0
PDMS	112.2 ± 2.9	102.6 ± 2.8	11.6 ± 2.6

Supplementary Table 2. Surface energies of the liquids used for the contact angle measurements (mN/m)

Tested liquid	Total	Dispersion coefficient	Polarity coefficient
Water	72.8	22.1	50.7
Glycerol	63.6	37.2	26.4

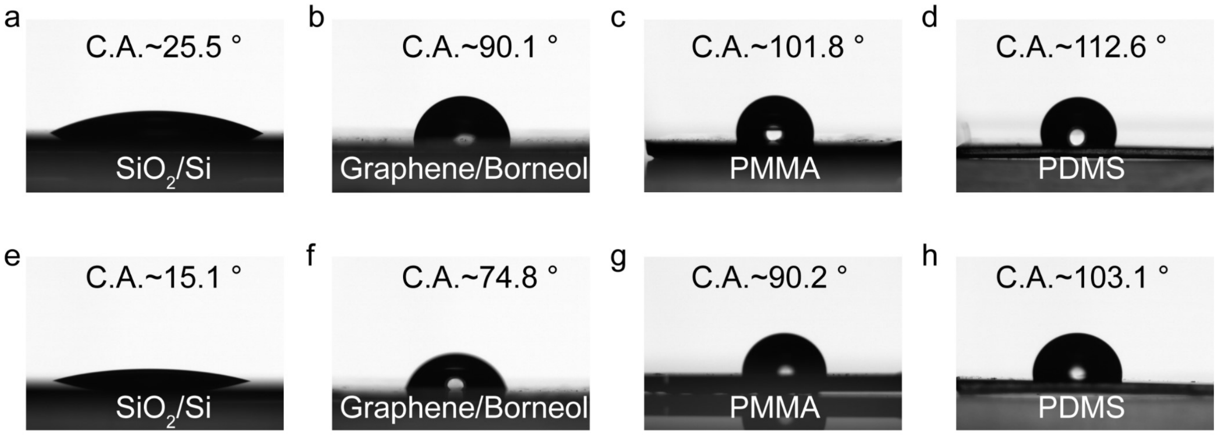

Supplementary Figure 4. The images of contact angles of tested liquids on different surfaces. (**a-d**) Images of contact angles of water on different substrates, (**e-h**) Images of contact angles of glycerol on different substrates. C. A. is the abbreviation of contact angle. The contact angle of water gradually increases from SiO_2/Si to PDMS, indicating the decrease of the surface energy.

Section III: The Gradient surface energy (GSE) strategy enables the intact and clean transfer of ultra-flat wafer-scale graphene (Supplementary Figure 5-14)

Mechanism of gradient surface energy modulation

The wettability can affect adhesion behavior. Incomplete wetting will produce interfacial defects and thereby lower the adhesive bond strength, whereas better wetting can increase the work of adhesion, which is directly proportional to the fracture energy. When an adhesive is applied on an adherend, many microscopic unwetted voids will be formed at the interface, as real surfaces are rarely perfectly smooth. The driving force for the wetting of these interfacial voids is the spreading coefficient λ_{AB}:

$$\lambda_{AB} = \gamma_B - \gamma_A - \gamma_{AB} \quad (4)$$

where λ_{AB} is the spreading coefficient of phase A (the adhesive) on phase B (the adherend); γ_B and γ_A are the surface energies of phase B and A, respectively; γ_{AB} is the interface energy of phase A and phase B. The fracture strength σ_f of the interface is related to λ_{AB} by[2]:

$$\sigma_f = \frac{K_m}{1-\left(\frac{\lambda_{AB}}{\gamma_B}\right)} = \frac{K_m \gamma_B}{\gamma_A + \gamma_{AB}} \quad (5)$$

Where K_m is a function of the mechanical properties. Since $\gamma_{AB} \ll \gamma_A$, the fracture strength is proportional to the surface energy ratio of the adherend to the adhesive.

The surface energy of SiO_2/Si (γ_1) was much larger than that of graphene/borneol (γ_2), which should greatly facilitate the reliable adhesion of graphene to the SiO_2/Si wafer. It is because that the low-surface-energy materials tend to adsorb strongly on large-surface-energy materials, resulting in better wettability. Moreover, the surface energy of PDMS (γ_4) was very small and close to the surface energy of PMMA (γ_3), ensuring the intact release of wafer-scale graphene onto the SiO_2/Si wafer due to the weak fracture strength of interface (Supplementary Figure 5). By comparison, the thermal release tape (TRT, REVALPHA, Nitto) had a stickier surface and much larger surface energy than that of PDMS, resulting in a poor release of wafer-scale graphene due to the uncontrolled expansion process at high temperature and large adhesion between TRT and PMMA (Supplementary Figure 6).

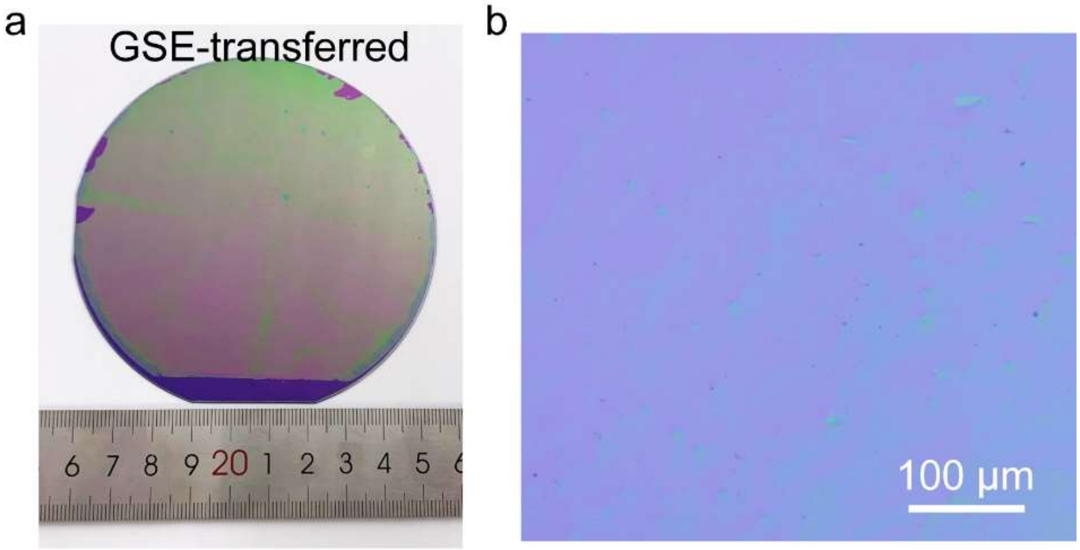

Supplementary Figure 5. Gradient surface energy enables the intact release of wafer-scale graphene. (**a**) Optical picture of GSE-transferred graphene after removing PDMS. (**b**) Optical microscopy (OM) images of PMMA/borneol/graphene on SiO_2/Si. The macroscopic and microscopic integrity of transferred graphene were ensured by intact release of wafer-scale graphene.

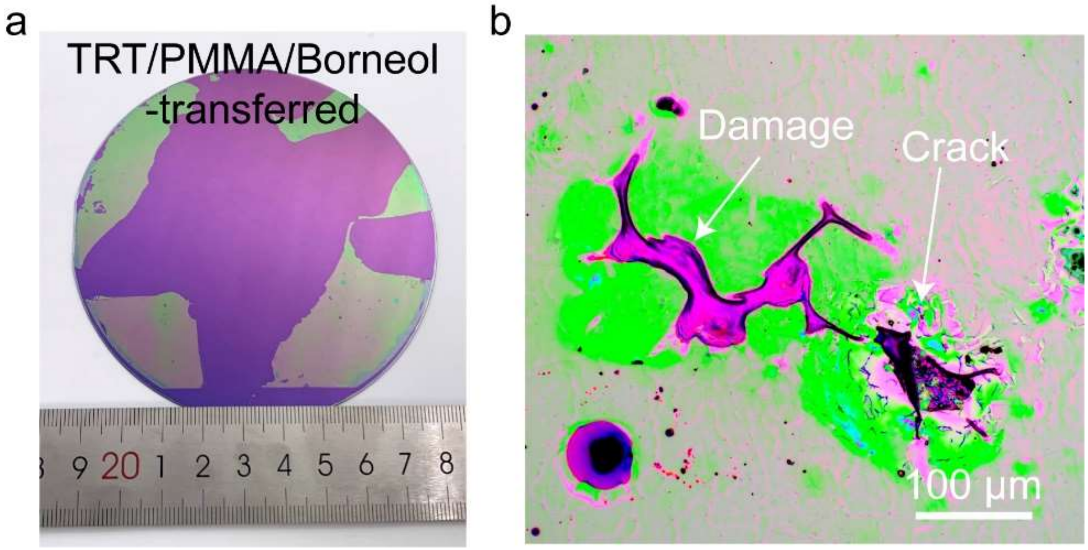

Supplementary Figure 6. Integrity of graphene suffers from the uncontrolled release process. (**a**) Optical picture of TRT/PMMA/Borneol-transferred graphene after removing TRT. (**b**) Optical microscopy (OM) images of PMMA/Borneol/graphene on SiO_2/Si, large damage and cracks caused by the sticky surface of TRT and uncontrolled expansion process at high temperature can be seen. TRT expanded and deformed during the heating, thus leading to the morphological defects in graphene.

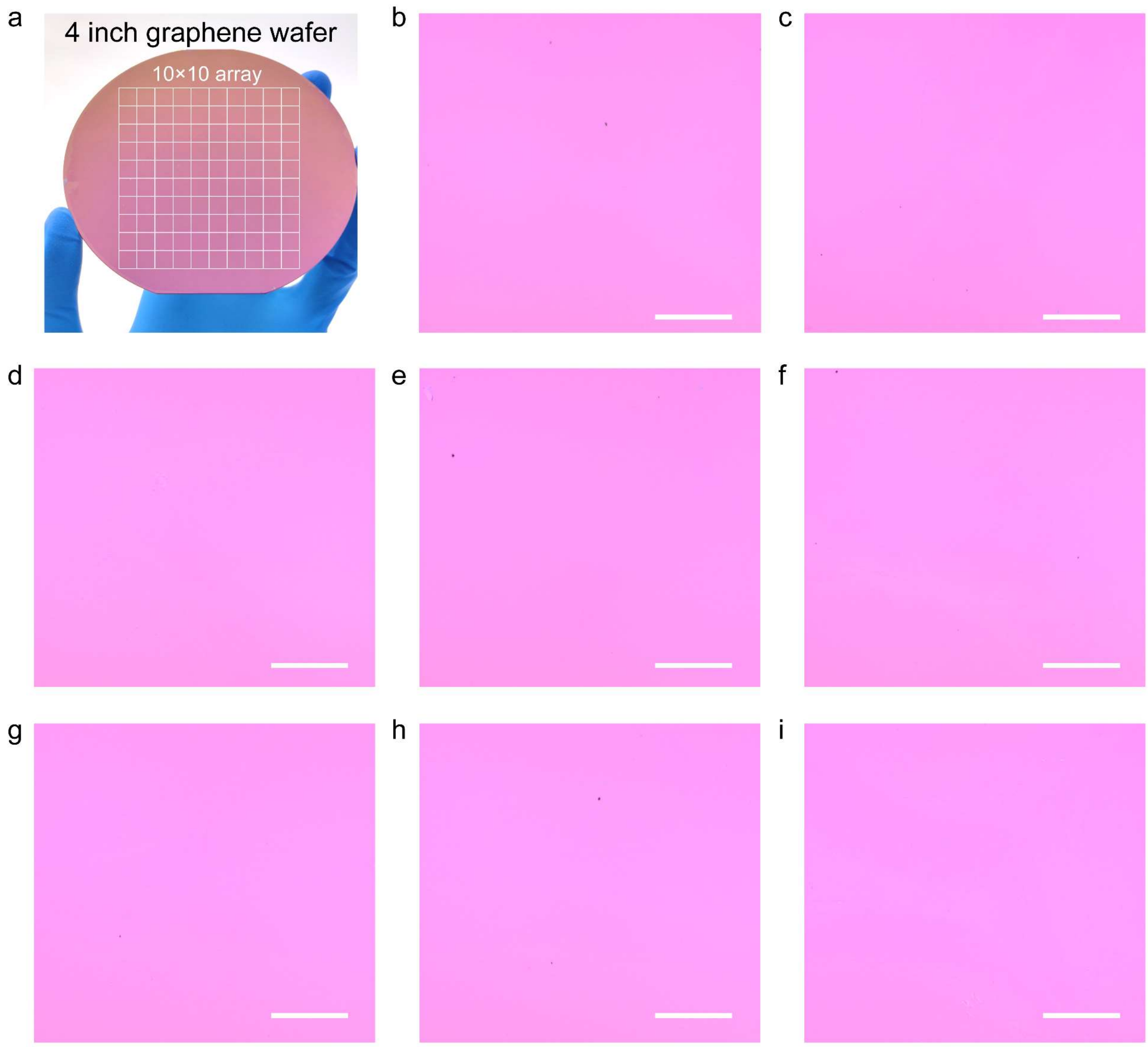

Supplementary Figure 7. Optical microscopy images of transferred graphene. (**a**) Optical picture of GSE-transferred graphene. (**b-i**) Typical optical microscopy (OM) images of GSE-transferred graphene from the marked area in (**a**) with a 10× objective, the scale bar is 200 μm. The surface of GSE-transferred graphene was almost free of structural defects and polymer residues.

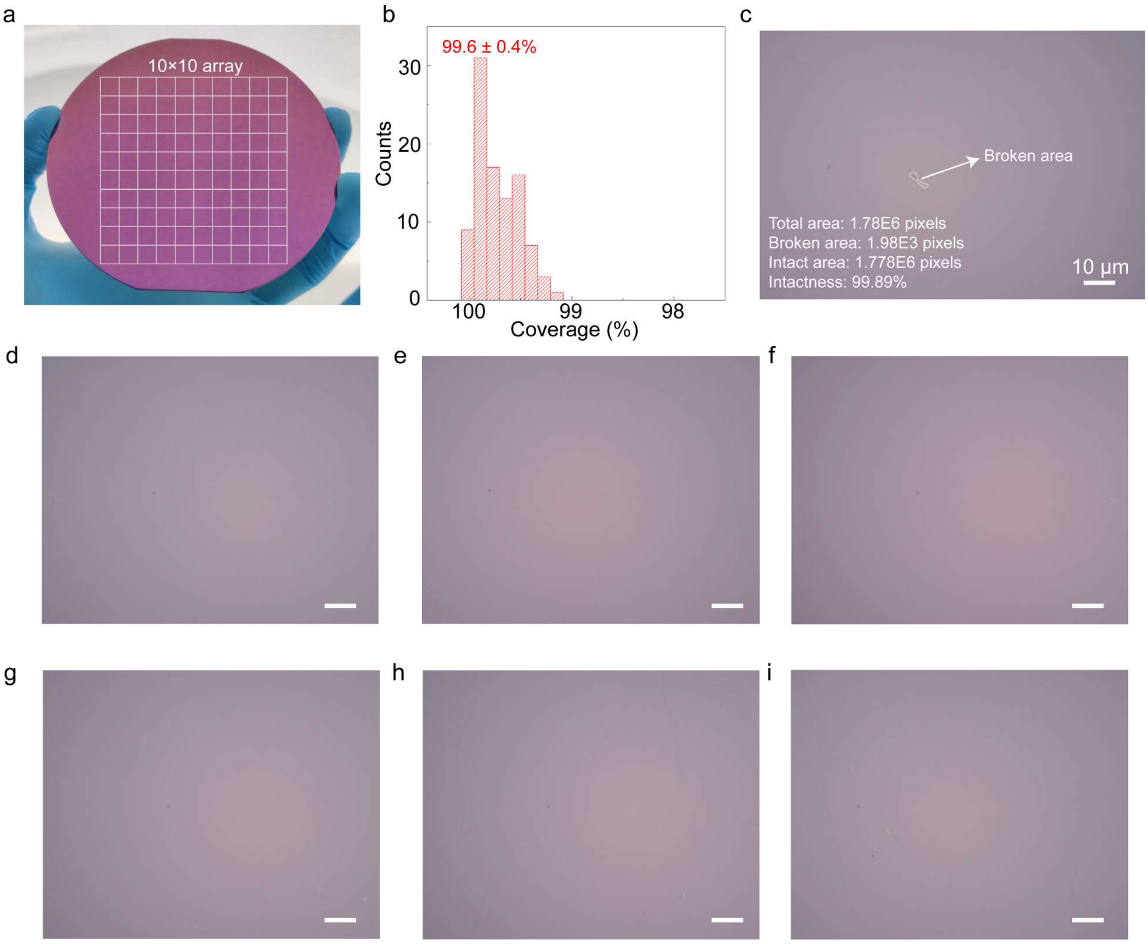

Supplementary Figure 8. Analysis of micro-intactness of transferred graphene. (**a**) Optical image of a 4-inch GSE-transferred graphene film on the SiO_2/Si wafer. (**b**) The histogram of graphene coverage analyzed by 100 optical microscopy images at 100× magnification from the marked area in (**a**). (**c**) One optical microscopy image of transferred graphene. The broken area is indicated by white dashed line. (**d-i**) Typical OM images of GSE-transferred graphene with 100× objective. Note that the optical image was processed with white balance to highlight microscopic damage. The scale bar is 10 μm.

We captured 100 optical microscopy images arranged in a 10×10 array over the 4-inch GSE-transferred graphene film (Supplementary Figure 7a). The area of image captured with a 10× objective is about 0.7 cm^2, so the total area of Figure 1e we analyzed is about 70 cm^2 which is 86% of the whole 4-inch area. We also collected 100 optical microscopy images arranged in a 10×10 array at 100× magnification (Supplementary Figure 8a), and the average coverage of transferred graphene is 99.6 ± 0.4% (Supplementary Figure 8b). The coverage is calculated by

$$\text{Coverage} = 1 - (\text{broken area}/\text{total area})$$

In details, the pixels of whole picture, broken area and intact area are 1.78×10^6, 1.98×10^3 and 1.778×10^6, respectively. So, the coverage equals pixels of intact area divided by pixels of total area (Supplementary Figure 8c). Typical optical images can be found in Supplementary Figure 8d-i.

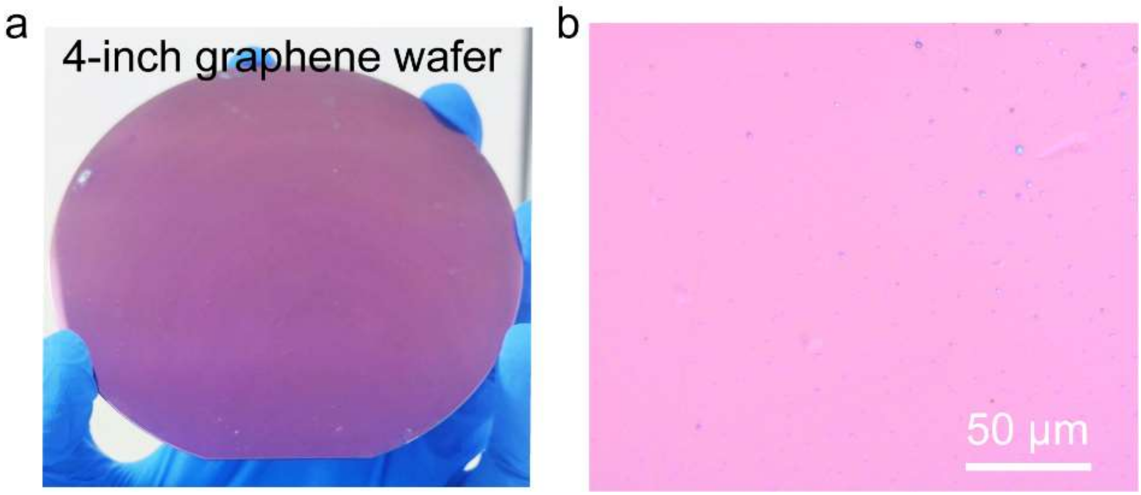

Supplementary Figure 9. Typical results of PMMA-transferred graphene. (**a**) Optical picture of PMMA-transferred graphene. (**b**) Typical OM image of PMMA-transferred graphene on SiO_2/Si with a 50x objective. Damages and polymer residues can be seen in PMMA-transferred graphene.

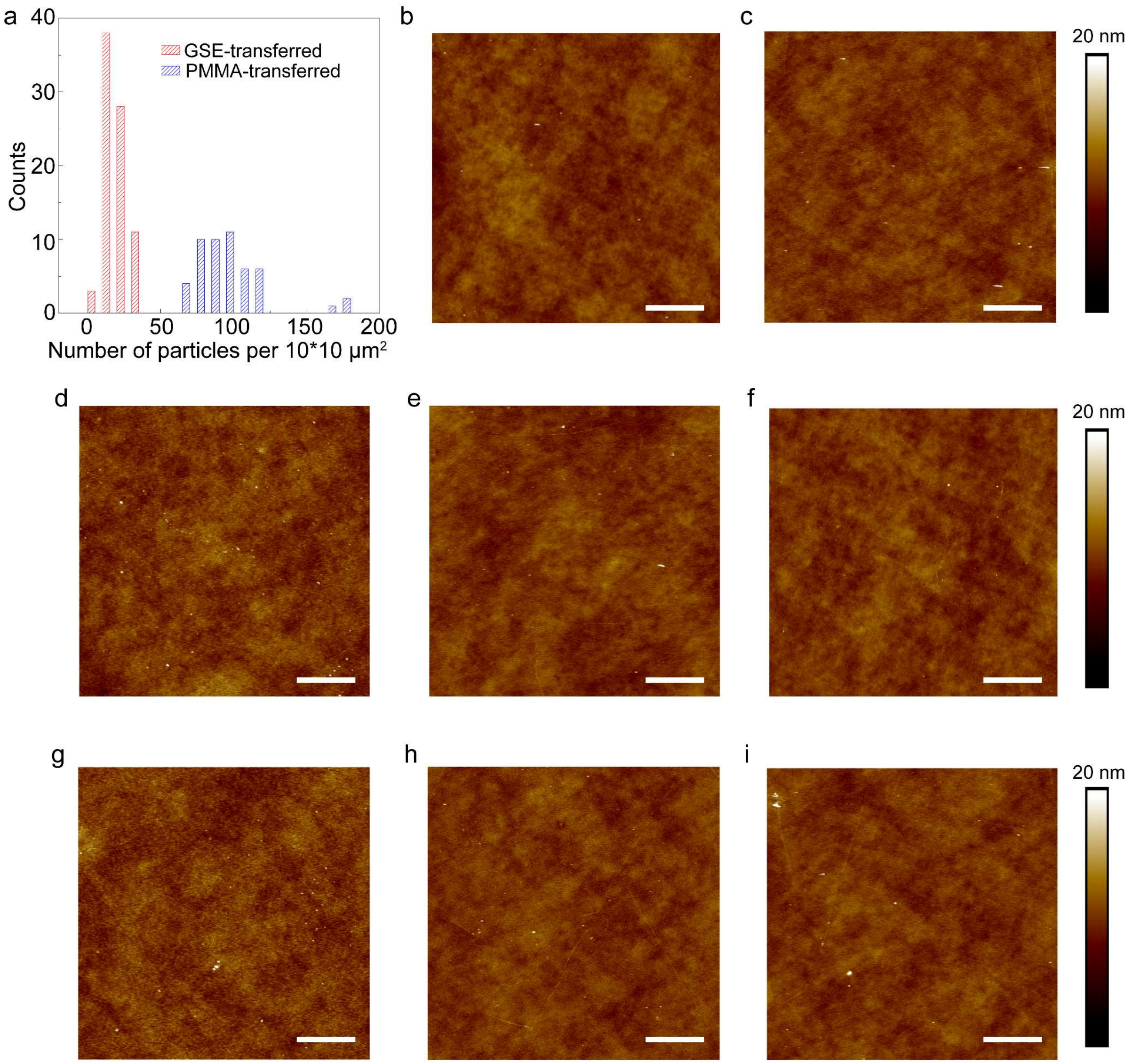

Supplementary Figure 10. Histograms of particle number and typical AFM images of GSE-transferred graphene. (**a**) Histograms of particle number per 10×10 μm^2 from 80 AFM images of GSE-transferred graphene and 50 AFM images of PMMA-transferred graphene. (**b-i**) Typical AFM images of GSE-transferred ultra-flat graphene. The scale bar is 2 μm.

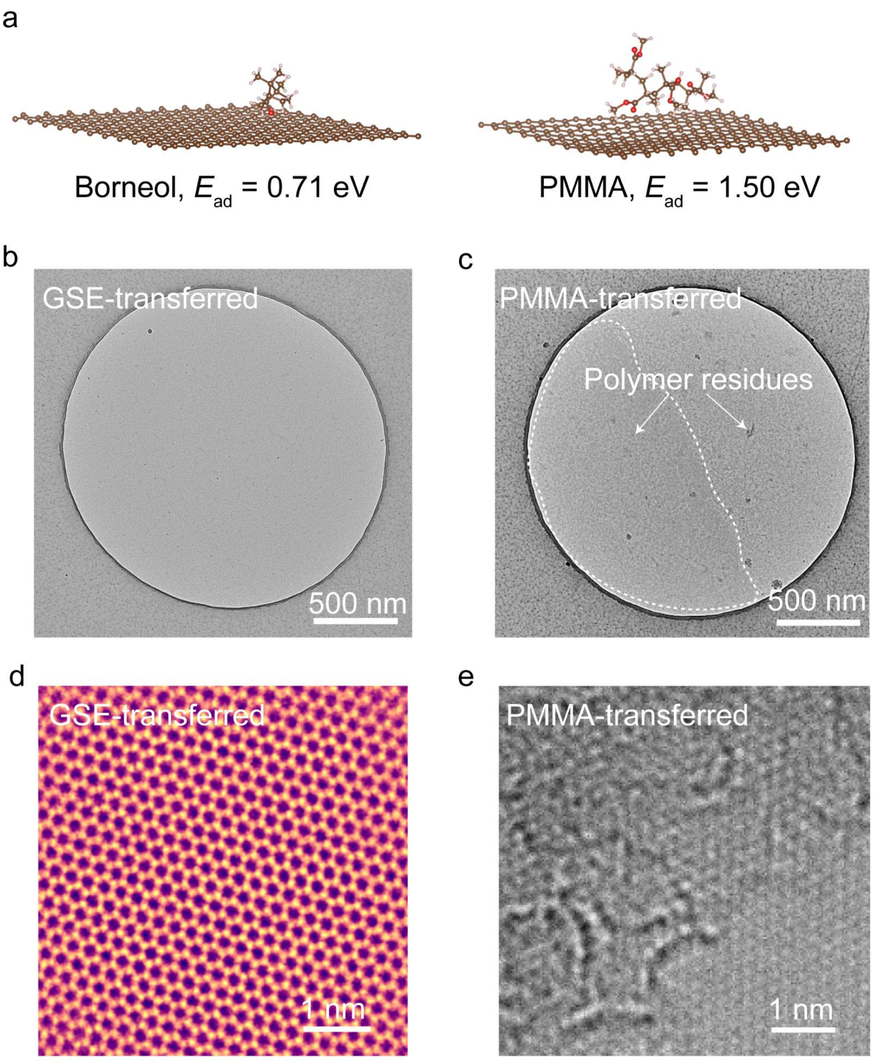

Supplementary Figure 11. Comparison of adsorption energies of borneol and PMMA and typical transmission electron microscopy (TEM) images of GSE- and PMMA-transferred graphene. (**a**) The adsorption energies of borneol and PMMA on graphene. (**b-c**) Typical low magnification TEM images of GSE- and PMMA-transferred graphene. The adsorption energy of borneol on graphene was approximately one half of that of PMMA, leading to a cleaner surface with a clearly atomic image. (**d-e**) Typical HRTEM images of GSE-transferred (**d**) and PMMA-transferred graphene (**e**).

The small molecule (borneol) demonstrates a lower adsorption energy on graphene than that of PMMA (Supplementary Figure 11a), leading to a clean graphene surface. As shown in Supplementary Figure 7-11, the GSE-transferred graphene had a cleaner surface than that of PMMA-transferred ones, whose surface was covered with dense polymer contaminations. Many polymer residues were adsorbed on the surface of PMMA-transferred graphene (Supplementary Figure 11c), and almost no clean area was observed (Supplementary Figure 11e). In contrast, no obvious polymer residue was observed on the GSE-transferred graphene (Supplementary Figure 11b), and the graphene lattice can be well characterized (Supplementary Figure 11d).

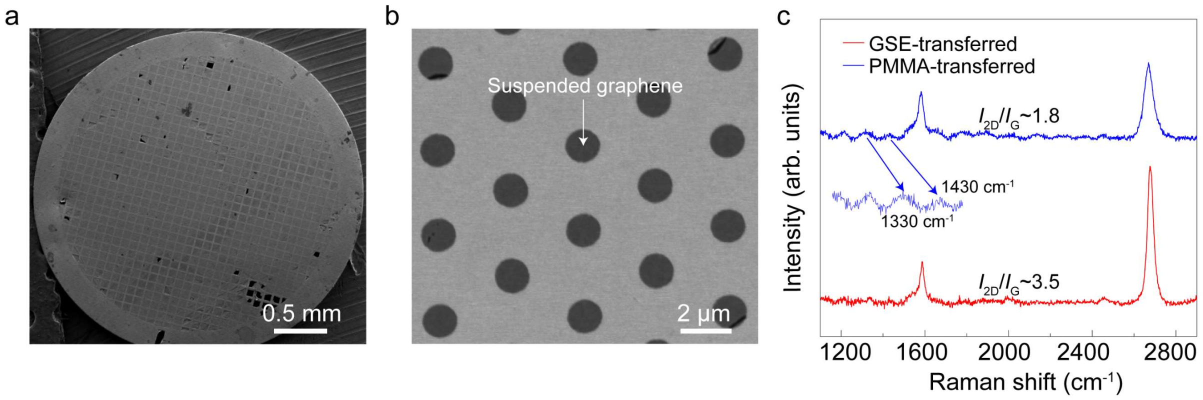

Supplementary Figure 12. Raman spectra of suspended GSE- and PMMA-transferred graphene. (**a**) SEM image of GSE-transferred graphene on Au TEM grid. (**b**) Typical SEM image of suspended graphene membranes. (**c**) The Raman spectra of GSE- and PMMA-transferred suspended graphene. Inset: fine spectra of PMMA-transferred graphene from 1100 to 1500 cm^{-1}.

Acquiring the Raman spectrum of graphene in a freestanding state is a reliable way to show the cleanliness of transferred graphene. Without the interference of substrate, polymer residues can be revealed near the D peak. As shown in Supplementary Figure 12, two obvious peaks at 1330 cm^{-1} and 1430 cm^{-1} appeared in the PMMA-transferred suspended graphene (Supplementary Figure 12c), corresponding with the previous reported results[3]. In contrast, no peaks were observed near the D band of GSE-transferred suspended graphene. Besides, the intensity ratio of 2D peak to G peak (I_{2D}/I_G) is informative of impurities on the graphene surface[4]. The I_{2D}/I_G of GSE-transferred graphene is ~3.5, much higher than that of PMMA-transferred graphene (~1.8), indicating the clean graphene surface with few impurities.

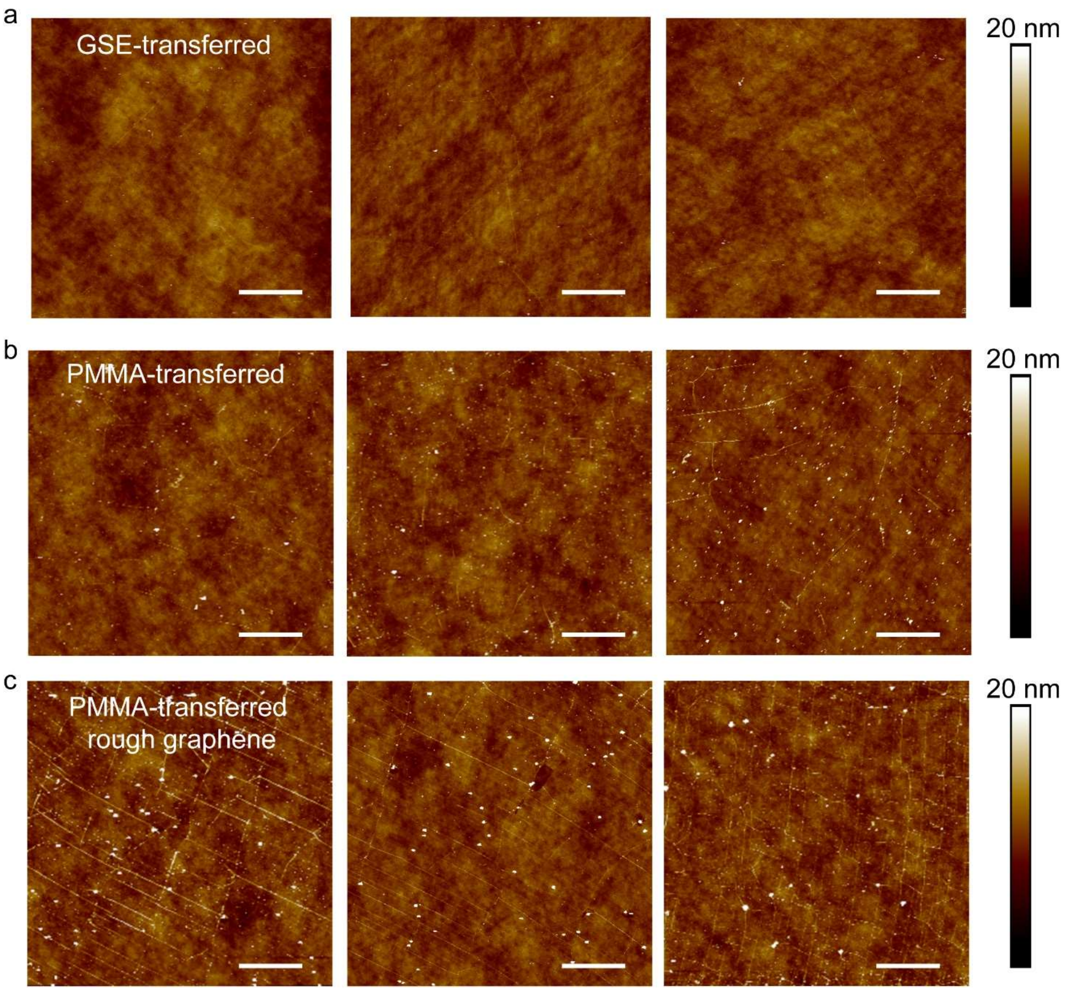

Supplementary Figure 13. Comparison of typical atomic force microscopy (AFM) images of transferred graphene. (**a, b**) AFM images of GSE- (**a**) and PMMA-transferred (**b**) ultra-flat graphene. There was almost no polymer residue on the surface of GSE-transferred graphene whereas many large residues can be found on the surface of PMMA-transferred graphene. (**c**) AFM images of PMMA-transferred rough graphene. The density of wrinkle number of transferred rough graphene was much larger than that of ultra-flat graphene, which should largely hinder the achievement of high-performance graphene electronic devices. The scale bar is 4 μm.

As shown in Fig. 1g, Supplementary Figure 10 and Supplementary Figure 13a-b, the particle number (~95) of PMMA-transferred graphene was larger than that of GSE-transferred graphene (~20). Moreover, it is worthy to note that the wrinkle number of transferred ultra-flat graphene was much smaller than that of transferred rough graphene (Fig. 1h and Supplementary Figure 13c) due to the wrinkle number of transferred graphene is related to the roughness of graphene on growth substrate (Supplementary Figure 14).

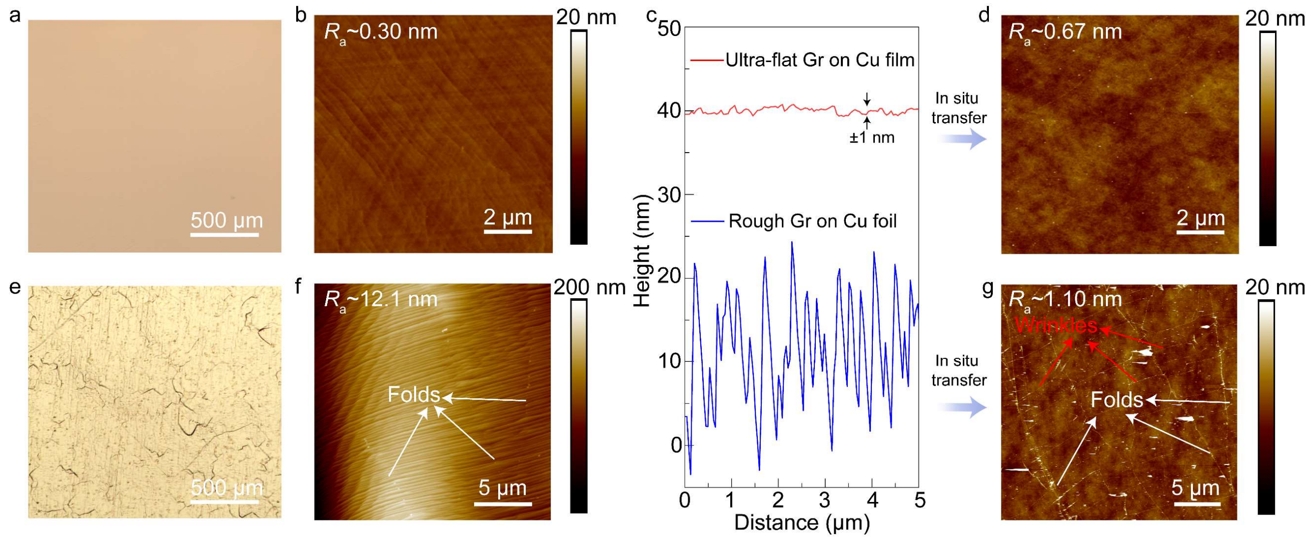

Supplementary Figure 14. The relationship between the roughness and wrinkle density of transferred graphene. (**a-b**) OM and AFM images of ultra-flat graphene grown on Cu(111) film. (**c**) Height profile of ultra-flat graphene on Cu film (**b**) and rough graphene on Cu foil (**f**). (**d**) The AFM image of in situ transferred graphene in (**b**). There were few folds and step bunches in ultra-flat graphene, and the surface roughness (R_a) of graphene in (**b**) was 0.30 nm, leading to a smooth graphene surface after transfer (**d**). (**e-f**) OM and AFM images of rough graphene grown on Cu(111) foil. (**g**) The AFM image of in situ transferred graphene in (**f**). The surface roughness of rough graphene (**f**) was 12.1 nm with several folds (white arrows) and many Cu step bunches. Step bunches will turn into new wrinkles (red arrows) after transfer (**g**).

Graphene wrinkles and Cu step bunches are usually formed during the growth of graphene, due to the mismatch of thermal expansion coefficient between graphene and the underlying Cu substrate[5]. To minimize the adverse effects of wrinkles on the charge carrier mobility, ultra-flat graphene films were grown on the 4-inch Cu(111)/sapphire wafers for graphene transfer. There are few wrinkles in the graphene grown on the Cu(111)/sapphire wafer (Supplementary Figure 14a-b), owing to the small thermal expansion of the Cu(111) thin film (~500 nm in thickness) on sapphire and the relatively strong interfacial coupling between Cu(111) and graphene[6].

Moreover, the height of Cu step of ultra-flat graphene/Cu(111)/sapphire is only ~2 nm, which is significantly smaller than that of copper foil (~20 nm) with dense Cu step bunches (Supplementary Figure 14c). We found that the step bunches of copper foil will cause the formation of new wrinkles after the transfer, revealed by our in situ transfer process. As shown in Supplementary Figure 14f, three folds and dense step bunches were observed on the graphene grown on the copper foil. After the transfer, the folds did not disappear, and new wrinkles appeared along the direction of step bunches (Supplementary Figure 14g). Since the step bunches were largely inhibited on the ultra-flat graphene grown on the Cu(111)/sapphire, the transfer-induced wrinkles can be significantly reduced (Supplementary Figure 14d).

Section IV: The universality of GSE strategy (Supplementary Figure 15-18, Supplementary Movie 2)

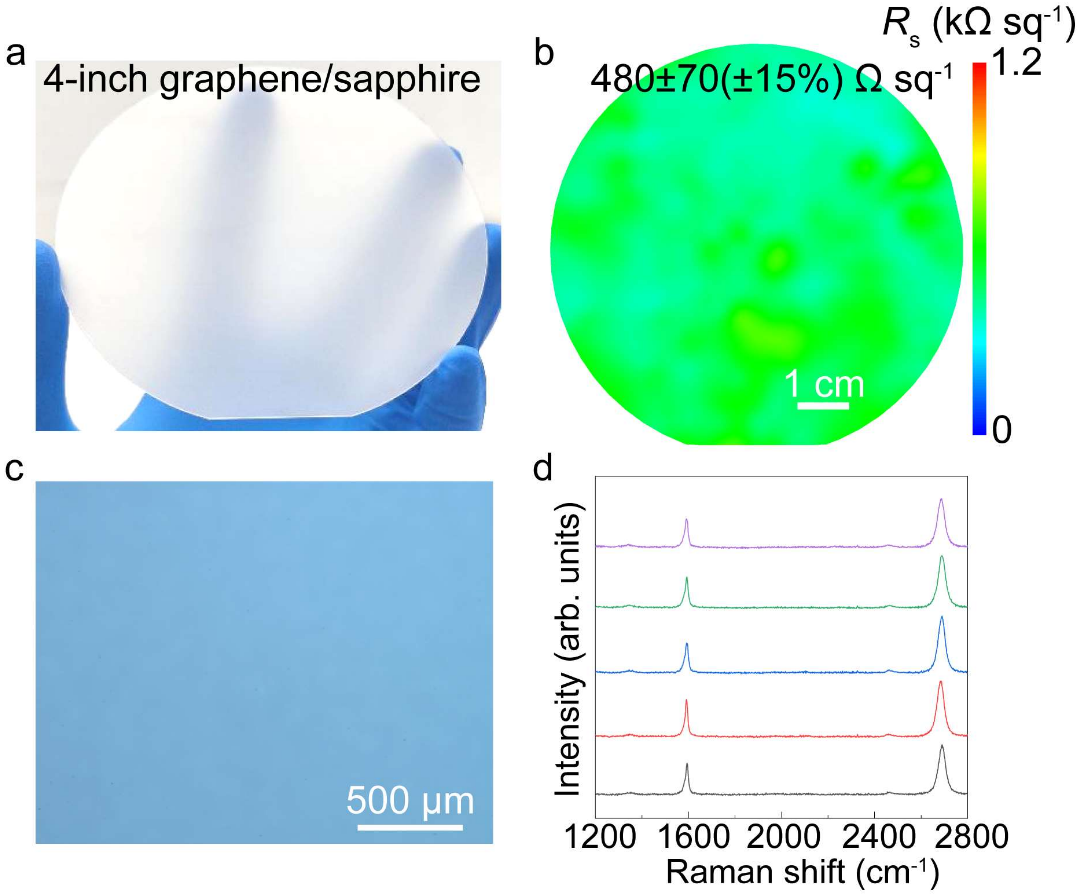

Supplementary Figure 15. Integration of ultra-flat graphene onto 4-inch sapphire wafer. (**a**) Optical picture of GSE-transferred graphene on sapphire wafer. (**b**) Spatial sheet resistance maps of GSE-transferred graphene on 4-inch sapphire. The sheet resistance deviation was ~15%. (**c**) Typical OM image of transferred graphene on sapphire. (**d**) The typical Raman spectra of transferred graphene.

The GSE strategy can also achieve the integration of graphene on sapphire, which can be used as a buffer layer for III-V semiconductor growth[7,8]. As shown in Supplementary Figure 15a, b, the GSE-transferred graphene had a very uniform sheet resistance (480 ± 70 Ω sq^{-1}), whose standard deviation was only ~15% over 4-inch sapphire wafer. The surface of transferred graphene was clean without polymer residues (Supplementary Figure 15c). No D band was observed in Raman spectra of transferred graphene (Supplementary Figure 15d).

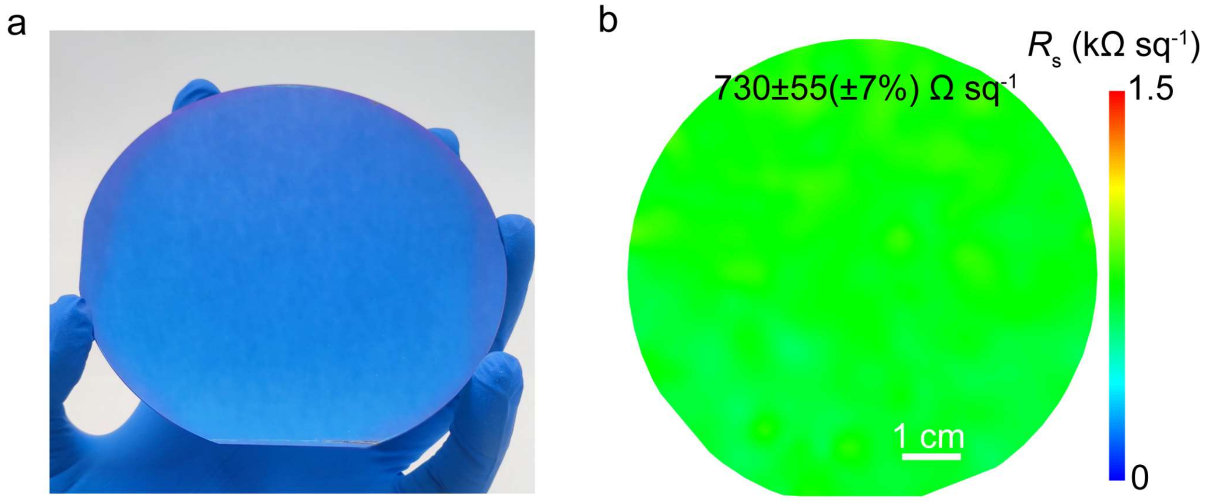

Supplementary Figure 16. Transfer of graphene grown on Cu foil onto SiO_2/Si. (**a**) Optical image of GSE-transferred graphene on SiO_2/Si. (**b**) Spatial sheet resistance map of transferred graphene with a ~7% deviation over a 4-inch area.

In addition, GSE method can also be used to transfer graphene grown on Cu foil, which was also recorded in Supplementary Movie 2. And the obtained graphene showed a uniform sheet resistance with a small deviation of ~7% as shown in Supplementary Figure 16.

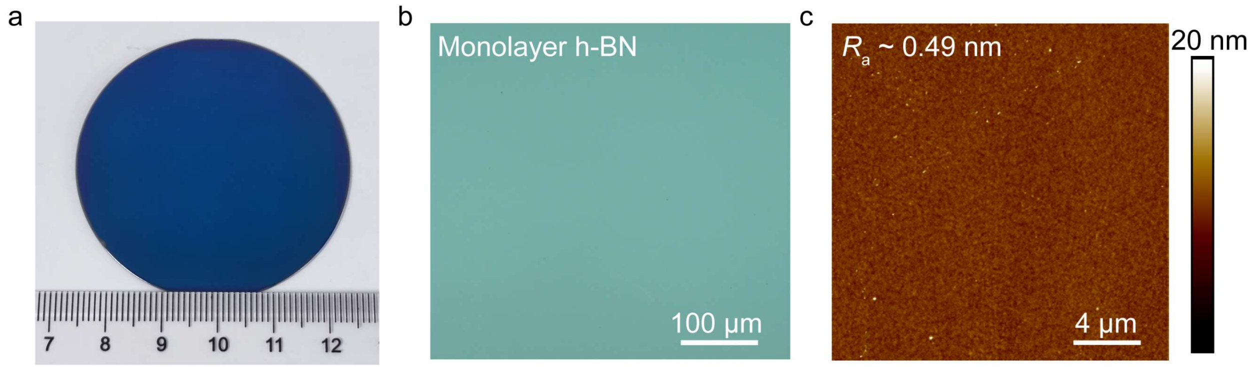

Supplementary Figure 17. Wafer-scale h-BN transferred using GSE method. (**a**) optical image of GSE-transferred 2-inch h-BN film. (**b**) Optical microscopy image of transferred h-BN with 20× objective. (**c**) AFM image of transferred h-BN, yielding a small surface roughness of ~0.49 nm.

h-BN is an important 2D material that can act as a buffer layer for the deposition of dielectric layer to construct photoelectronic devices[9] and an encapsulation layer of graphene for the achievement of high-performance graphene electronic devices[10]. Wafer-scale single crystal monolayer h-BN can be synthesized on metal substrate[11,12] and can be integrated to target substrates using our GSE strategy (Supplementary Figure 17), which should further facilitate the application of h-BN in electronics and optoelectronics.

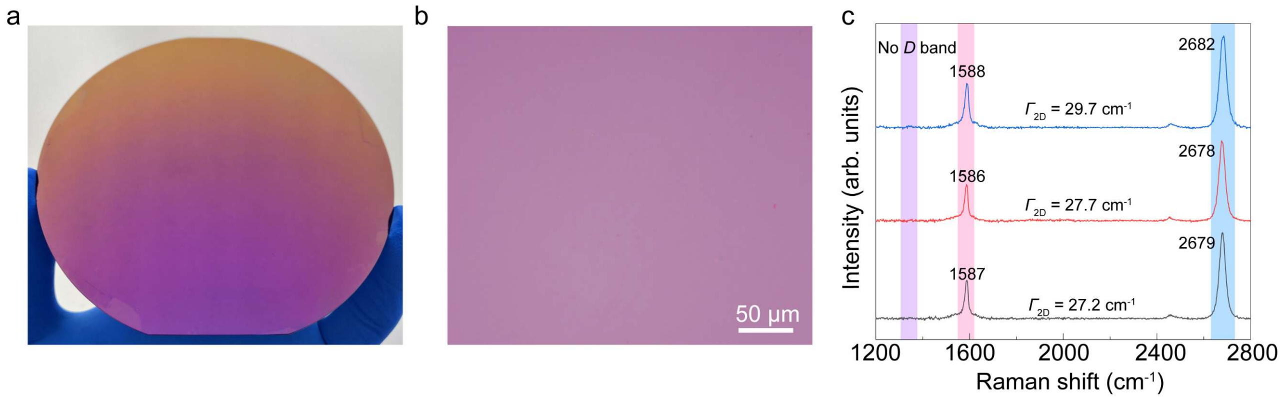

Supplementary Figure 18. GSE transfer results using rosin as a small molecule buffer layer. (**a**) Optical image of 4-inch GSE-transferred graphene. (**b**) Typical optical microscopy image of graphene at 20× magnification. (**c**) Typical Raman spectra of GSE-transferred graphene on the SiO_2/Si wafer.

We achieved similar results using the same GSE method by replacing borneol with rosin, a small natural organic molecule that has a weak interaction with graphene[13]. As shown in Supplementary Figure 18, the wafer-scale GSE-transferred graphene is intact and clean. No D band can be seen in Raman spectra (Supplementary Figure 18c), and the FWHM of 2D peak is ~28 cm^{-1}, indicating the GSE-transferred graphene has little random strain fluctuation and potentially high charge carrier mobility.

Section V: Raman spectra of GSE-transferred graphene (Supplementary Figure 19)

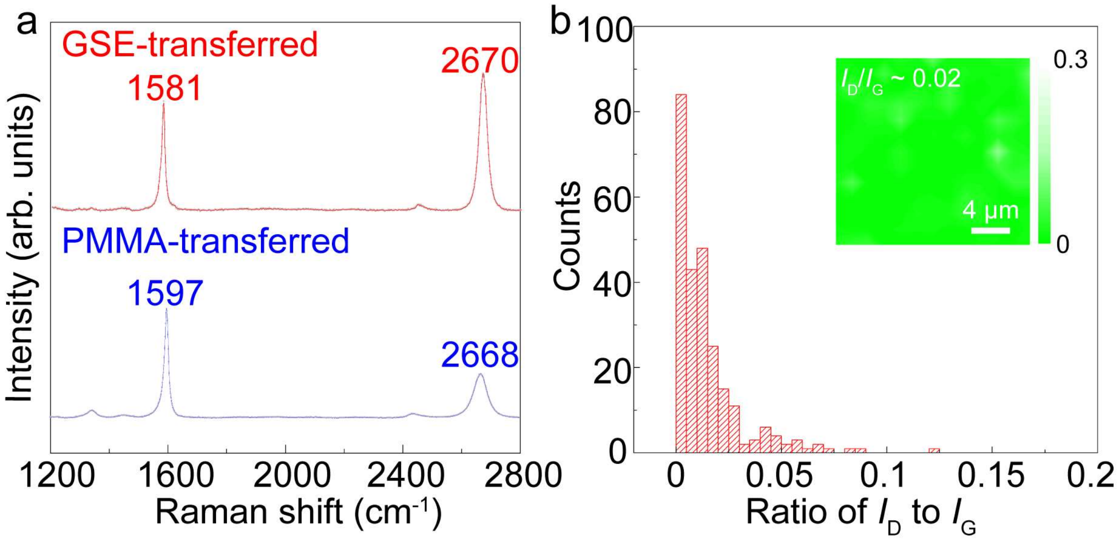

Supplementary Figure 19. Raman spectra of GSE-transferred graphene on SiO_2/Si. (**a**) Typical single point Raman spectrum of GSE- (Red line) and PMMA-transferred (Blue line) graphene, respectively. No D band can be seen in the GSE-transferred graphene, and the blue-shifted G peak position of PMMA-transferred graphene indicated a larger doping level. (**b**) Histogram of I_D/I_G ratio of GSE-transferred graphene with spatial map of I_D/I_G ratio (Inset).

As shown in Supplementary Figure 19a, the position of G band and 2D band of PMMA-transferred graphene were blue shifted compared to GSE-transferred graphene, indicating a higher doping level of graphene[14]. The doping is always caused by water at the interface and polymer residue on the surface, limiting the carrier mobility and device performance. The concentration of defect in graphene can also affect its electrical properties. As shown in Supplementary Figure 19b, the ratio of the amplitude of D band to the G band was about 0.02, indicating a low point defect concentration[15].

Section VI: Electrical performance of transferred graphene on SiO_2/Si (Supplementary Figure 20-22)

To evaluate the electronic property, the monolayer single-crystalline graphene was transferred onto the 250 nm SiO_2/Si substrate for the fabrication of Hall-bar devices by using standard electron beam lithography (EBL). The four-terminal electrical measurements on the Hall bars were carried out at room temperature and the charge carrier mobility μ was extracted from linear fits of the conductivity σ via equation[16] (6):

$$\mu_{4\text{p}} = \left(\frac{L}{W C_{ox} \Delta V} \right) \left(\frac{dI_{ds}}{dV_g} \right) \quad (6)$$

Where C_{ox} is the gate capacitance (1.38×10^{-8} F cm^{-2} for 250 nm thick SiO_2); L and W are the spacing between the inner edges of the voltage probes and channel width, respectively; I_{ds} is the drain-to-source current; ΔV is the voltage drop between the two probes separated by L; and V_{g} is the back-gate voltage.

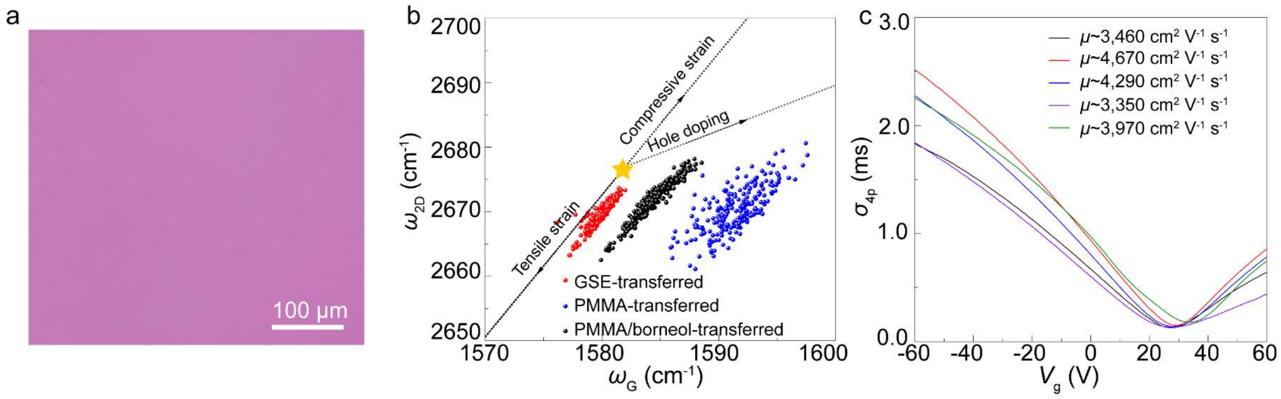

Supplementary Figure 20. Properties of PMMA/borneol-transferred graphene. (**a**) Typical optical microscopy image of PMMA/borneol-transferred graphene on SiO_2/Si with a 20× objective. (**b**) Correlation map of the Raman G and 2D peak positions of PMMA/borneol-transferred graphene comparing to GSE- and PMMA-transferred graphene. The yellow star represents the G and 2D peak positions of the pristine graphene with neither doping nor strain. (**c**) Transfer characteristics of 5 typical Hall-bar devices fabricated with PMMA/borneol-transferred graphene.

If the PMMA/borneol was removed with acetone instead of laminating PDMS, we can also get intact and clean graphene as shown in Supplementary Figure 20a. However, the doping level of that graphene was still larger than GSE-transferred mainly due to the water-adsorption-induced doping at the interface when graphene was scooped on substrate in water (Supplementary Figure 20b). The electrical performances of devices fabricated with such graphene films were also investigated. The typical transfer characteristics of 5 Hall-bar devices fabricated with this graphene are summarized in Supplementary Figure 20c. The Dirac point of graphene was close to 29 V, and the carrier concentration was relatively large (~2×10^{12} cm^{-2}), showing a low carrier mobility of ~3,950 cm^2 V^{-1} s^{-1}. The water at the interface deeply increased the doping level of graphene and decreased the electrical performance of graphene devices. Therefore, PDMS layer is used and serves as a self-supporting layer, allowing the dry transfer of wafer-scale graphene and preventing water-adsorption-induced doping.

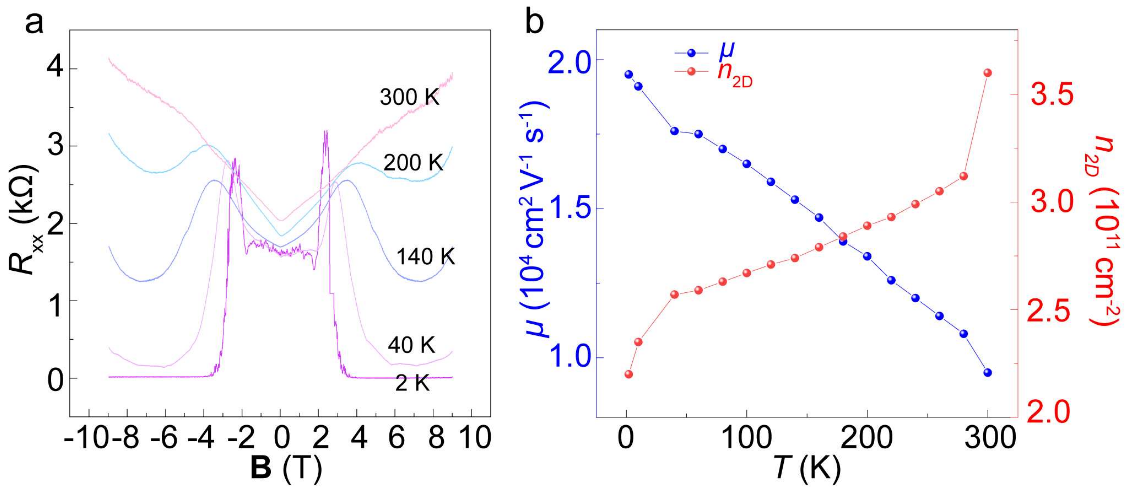

Supplementary Figure 21. Quantum Hall effect of GSE-transferred graphene on SiO_2/Si at room temperature. (**a**) Longitudinal resistance (R_{xx}) as a function of **B** at different temperatures from 2 K to 300 K. (**b**) Hall mobility and carrier concentration of GSE-transferred graphene at different temperature.

Quantum Hall effect (QHE) of transferred graphene on SiO_2/Si was observed at room temperature, indicating the transferred graphene is close to its intrinsic nature[17]. As shown in Supplementary Figure 21a, the Hall mobilities of the non-encapsulated graphene extracted from the Hall resistance (R_{xy})-magnetic fields (**B**) characteristic were 9,500 $cm^2\ V^{-1}\ s^{-1}$ and 19,500 $cm^2\ V^{-1}\ s^{-1}$ at room temperature and 2 K, respectively. By measuring the magnetoresistance (R_{xx}) and R_{xy} at different temperatures, we confirmed that the nonlinearity in the large magnetic field at room temperature was due to the quantum Hall effect. Furthermore, the carrier concentration was ~$3.5 \times 10^{11}\ cm^{-2}$ (Supplementary Figure 21b), indicating a lower doping level of GSE-transferred graphene.

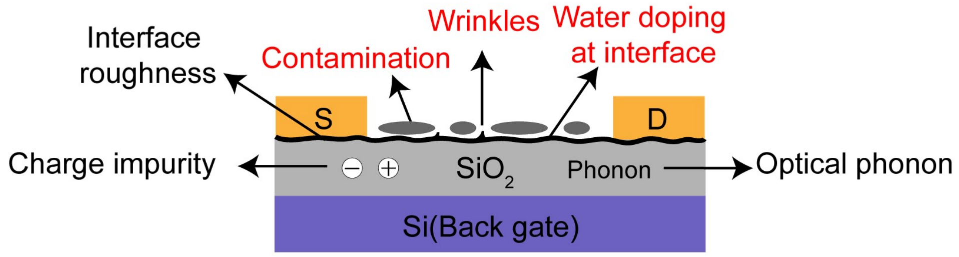

Supplementary Figure 22. Scattering factors affecting mobility of graphene. Scattering factors affecting mobility of graphene on SiO_2/Si.

The QHE can be observed at room temperature due to the highly unusual nature of charge carriers in graphene, which behave as massless Dirac fermions and move with little scattering under ambient conditions. There are several factors that help the QHE in graphene to survive to room temperature. One of the critical factors is ultra-high mobility, a mobility of ~10,000 $cm^2\ V^{-1}\ s^{-1}$ yields a scattering time of $\tau \sim 10^{-13}$ s, so that the high field limit $\omega_c\tau = \mu\cdot\mathbf{B} >> 1$ is reached in fields of several T[18].

The Hall mobility of GSE-transferred graphene on the SiO_2/Si was very high (~9500 $cm^2\ V^{-1}\ s^{-1}$), because the transferred graphene film was clean, ultra-flat and with negligible doping, indicating little scattering centers. Thus, we can observe the QHE at room temperature. In comparison, Y. P. Chen et al. can only observe the QHE in PMMA-transferred CVD graphene at a very low temperature (0.6 K), which can be attributed on the lower mobility (~3000 $cm^2\ V^{-1}\ s^{-1}$) caused by scattering centers, such as water doping, polymer contamination and wrinkles introduced by PMMA wet transfer process[19] (Supplementary Figure 22).

Beyond QHE, we also observed FQHE (Fig. 3g-h). To observe FQHE[20], the mobility of transferred graphene should be comparable to the high-quality exfoliated graphene with average mobilities exceeding 100,000 $cm^2\ V^{-1}\ s^{-1}$. GSE-transferred graphene had a clean, ultra-flat and negligible doping surface, which had an ultra-high mobility (~280,000 $cm^2\ V^{-1}\ s^{-1}$) after encapsulation by h-BN, rivaling mechanical exfoliated graphene. Thus, the observation of FQHE at 8.5 T, 1.7 K indicated an outstanding quality of our GSE-transferred graphene (Fig. 3g-h).

Section VII: Morphology and Raman peak position shift of graphene thermal emitter devices (Supplementary Figure 23)

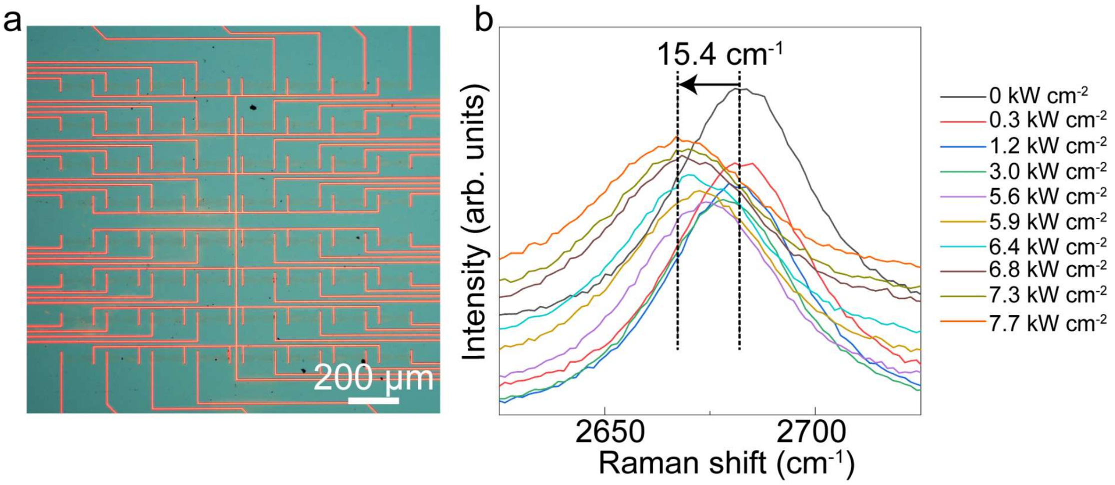

Supplementary Figure 23. Morphology and Raman peak position shift of graphene thermal emitter devices. (**a**) An array of 8×8 Al_2O_3-capped graphene emitters. (**b**) Raman 2D peaks of GSE-transferred graphene as a function of applied bias voltage. The temperature coefficient for the 2D peak in single layer graphene is -0.034 cm^{-1} K^{-1}.

To protect graphene, a ~70-nm-thick Al_2O_3 layer was deposited on graphene by atomic layer deposition before applying voltage (Supplementary Figure 23a). Under continuous DC bias voltage, the significant emission from the Al_2O_3-capped graphene device was observed with an Infrared (IR) camera. The graphene temperatures, which were obtained from the shift of 2D peak of biased graphene[21] (Supplementary Figure 23b), depended linearly on the power density. It can be attributed to the electron scattering with optical phonons caused by Joule heating[22] and the highest temperature can reach about 750 K at power density = 7.7 kW cm^{-2} in vacuum (Fig. 4f).

Supplementary References

1 Kotsidi, M. *et al.* Preventing colour fading in artworks with graphene veils. *Nat. Nanotechnol.* **16**, 1004-1010 (2021).

2 Wu, S. Polymer interface and adhesion (1st ed.), Routledge, (1982).

3 Lin, Y. C. *et al.* Clean transfer of graphene for isolation and suspension. *ACS Nano* **5**, 2362-2368 (2011).

4 Zhang, J. *et al.* Clean transfer of large graphene single crystals for high-intactness suspended membranes and liquid cells. *Adv. Mater.* **29**, 1700639 (2017).

5 Wang, M. *et al.* Single-crystal, large-area, fold-free monolayer graphene. *Nature* **596**, 519-524 (2021).

6 Deng, B. *et al.* Wrinkle-free single-crystal graphene wafer grown on strain-engineered substrates. *ACS Nano* **11**, 12337-12345 (2017).

7 Qi, Y. *et al.* Fast growth of strain-free AlN on graphene-buffered sapphire. *J. Am. Chem. Soc.* **140**, 11935-11941 (2018).

8 Chen, Z. *et al.* Improved epitaxy of AlN film for deep-ultraviolet light-emitting diodes enabled by graphene. *Adv. Mater.* **31**, e1807345 (2019).

9 Marconi, S. *et al.* Photo thermal effect graphene detector featuring 105 Gbit s^{-1} NRZ and 120 Gbit s^{-1} PAM4 direct detection. *Nat. Commun.* **12**, 806 (2021).

10 Wang, L. *et al.* One-dimensional electrical contact to a two-dimensional material. *Science* **342**, 614-617 (2013).

11 Wang, L. *et al.* Epitaxial growth of a 100-square-centimetre single-crystal hexagonal boron nitride monolayer on copper. *Nature* **570**, 91-95 (2019).

12 Chen, T.-A. *et al.* Wafer-scale single-crystal hexagonal boron nitride monolayers on Cu (111). *Nature* **579**, 219-223 (2020).

13 Zhang, Z. *et al.* Rosin-enabled ultraclean and damage-free transfer of graphene for large-area flexible organic light-emitting diodes. *Nat. Commun.* **8**, 14560 (2017).

14 Lee, J. E. *et al.* Optical separation of mechanical strain from charge doping in graphene. *Nat. Commun.* **3**, 1024 (2012).

15 Cançado L. G. *et al.* Quantifying defects in graphene via Raman spectroscopy at different excitation energies. *Nano Lett.* **11**, 3190-3196 (2011).

16 He, D. *et al.* Ultrahigh mobility and efficient charge injection in monolayer organic thin-film transistors on boron nitride. *Sci. Adv.* **3**, e1701186 (2017).

17 Yuan, G. *et al.* Proton-assisted growth of ultra-flat graphene films. *Nature* **577**, 204-208 (2020).

18 Novoselov, K. S. *et al.* Room-temperature quantum Hall effect in graphene. *Science* **315**, 1379-1379 (2007).

19 Cao, H. *et al.* Electronic transport in chemical vapor deposited graphene synthesized on Cu: Quantum Hall effect and weak localization. *Appl. Phys. Lett.* **96**, 122106 (2010).

20 Schmitz, M. *et al.* Fractional quantum Hall effect in CVD-grown graphene. *2D Materials* **7**, 041007 (2020).

21 Calizo, I. *et al.* Variable temperature Raman microscopy as a nanometrology tool for graphene layers and graphene-based devices. *Appl. Phys. Lett.* **91**, 071913 (2007).

22 Freitag, M. *et al.* Thermal infrared emission from biased graphene. *Nat. Nanotechnol.* **5**, 497-501 (2010).
